# Modelling evolution on design-by-contract predicts an origin of Life through an abiotic double-stranded RNA world

**DOI:** 10.1186/1745-6150-2-12

**Published:** 2007-04-27

**Authors:** Albert DG de Roos

**Affiliations:** 1 Syncyte BioIntelligence, PO Box 600, 1000 AP Amsterdam, The Netherlands

## Abstract

**Background:**

It is generally believed that life first evolved from single-stranded RNA (ssRNA) that both stored genetic information and catalyzed the reactions required for self-replication.

**Presentation of the hypothesis:**

By modeling early genome evolution on the engineering paradigm design-by-contract, an alternative scenario is presented in which life started with the appearance of double-stranded RNA (dsRNA) as an informational storage molecule while catalytic single-stranded RNA was derived from this dsRNA template later in evolution.

**Testing the hypothesis:**

It was investigated whether this scenario could be implemented mechanistically by starting with abiotic processes. Double-stranded RNA could be formed abiotically by hybridization of oligoribonucleotides that are subsequently non-enzymatically ligated into a double-stranded chain. Thermal cycling driven by the diurnal temperature cycles could then replicate this dsRNA when strands of dsRNA separate and later rehybridize and ligate to reform dsRNA. A temperature-dependent partial replication of specific regions of dsRNA could produce the first template-based generation of catalytic ssRNA, similar to the developmental gene transcription process. Replacement of these abiotic processes by enzymatic processes would guarantee functional continuity. Further transition from a dsRNA to a dsDNA world could be based on minor mutations in template and substrate recognition sites of an RNA polymerase and would leave all existing processes intact.

**Implications of the hypothesis:**

Modeling evolution on a design pattern, the 'dsRNA first' hypothesis can provide an alternative mechanistic evolutionary scenario for the origin of our genome that preserves functional continuity.

**Reviewers:**

This article was reviewed by Anthony Poole, Eugene Koonin and Eugene Shakhnovich

## Background

The evolution of life and the origins of DNA and RNA as the carriers of information are still a mystery. It has been proposed that the DNA world was preceded by an RNA world in which RNA fulfilled a role both as the information carrier and as the catalyst of early chemical processes [1,2]. The general idea is that from a pool of random strings of RNA, ribozymes would emerge with a primitive RNA polymerase activity and, in this way, RNA could provide its own replication machinery [3-8]. The transition from a ssRNA world to the dsDNA world is thought to have arisen by a reverse transcriptase activity that copied an RNA template to dsDNA, possibly by a dsRNA intermediate [9,10]. However, the abiotic availability of strands of RNA that are long enough to function as a ribozyme or exhibit replicase activity is uncertain. There are also no indications that ribozymes with polymerase activity have ever existed since the only naturally occurring ribozymes do not perform polymerization reactions [11,12]. The transition from ssRNA as the informational carrier to dsDNA is also mechanistically difficult since in order to maintain existing catalytic function, the concomitant evolution of a transcription system based on dsDNA is needed. Therefore, the fundamental molecular mechanisms that underlie the origin of life are still unknown.

The potential of RNA to perform as both an information carrier and as a catalytic molecule, is at the basis of the RNA world hypothesis. However, it is difficult to imagine ssRNA to have a dual function since the catalytic properties of RNA depend on the three-dimensional structure, while the informational capacity would require a simple linear structure that can be replicated [13]. The folding of an RNA molecule would prevent its own replication, while for replication a folded ribozyme would be necessary. Therefore, a dual function for the informational and the catalytic functions of ssRNA seem to be mutual exclusive on a biochemical mechanistic basis. From a system perspective, a dual function in a single entity would create a dependency that would reduce evolvability of the system, since each adaptation of one function could negatively affect the other function and would prevent an independent evolution. This is for instance apparent in the transition from ssRNA to dsDNA where the double-stranded form would completely prevent function until the development of a complex transcription machinery and would therefore not be based on the fundamental principle of functional continuity in evolution. Thus, a scenario that is not dependent on a dual function of ssRNA could be a starting point for alternative mechanisms for the origin of Life.

## The 'dsRNA first' hypothesis

An independent evolution of both informational and catalytic functionalities of RNA would provide the necessary flexibility for the evolution of the genome. The potential for system evolvability is met in software development by using the design-by-contract methodology that views a system as a set of communicating modules whose interaction is based on precisely defined interfaces. As long as existing interfaces stay intact, independent evolution of the individual modules is possible. In case of the genome, we can discern two separate functional modules: the ability to store genetic information on one hand, and the ability to function as a catalyst on the other hand. In all organisms, the informational and catalytic entities in the genome are well-separated into respectively dsDNA and ssRNA, and can therefore be seen as the mechanistic implementation of these functional modules. Their relation or interface is strictly defined and conserved throughout the Tree of Life: one part of the double-stranded DNA represents the template for the transcription of ssRNA. Thus, the separation of the informational and catalytic characteristics of RNA by a defined interface allows in principle an independent evolution of both functions as long as this interface stays intact.

**Figure 1:**
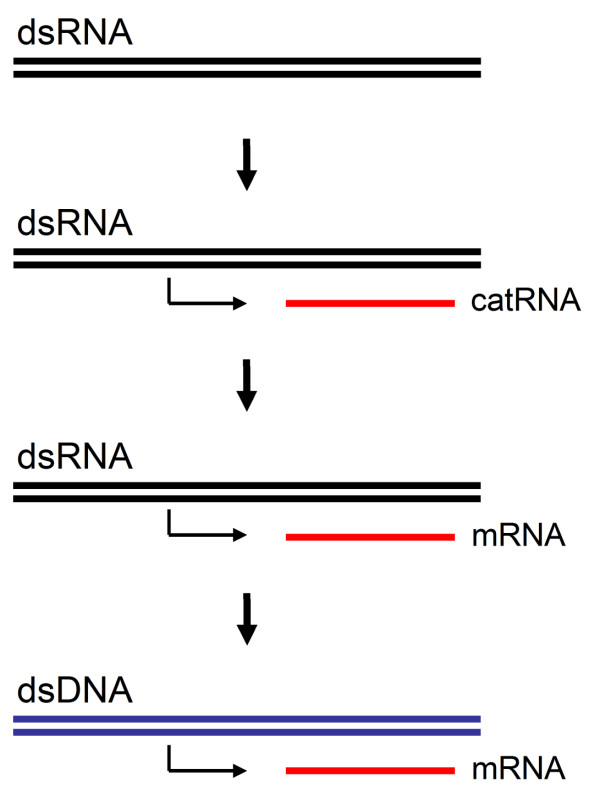
**The origin of genetic information carriers based on the conservation of existing interfaces**. The separation of informational and catalytic properties of RNA can be accomplished when dsRNA evolved first as the informational molecule, and was followed later in evolution by ssRNA as the catalytic molecule. In this scenario, catalytic RNA is derived from initial dsRNA and at a later stage, this ssRNA can then act as a the mRNA template for protein synthesis. A transition to dsDNA does not require a new kind of information carrier, but only the chemical adaptation of the existing one.

Design-by-contract predicts a scenario where once established interfaces remain intact during evolution and thus enforces functional continuity [14,15]. Since a double-stranded RNA provides the template and thus the functional interface for the derivation of single-stranded catalytic RNA, a straightforward explanation for the origin of catalytic RNA is when double-stranded RNA would also have preceded the catalytic single-stranded RNA in evolution. In this way, double-stranded RNA carried the informational function consistently from the start, while ssRNA always harboured the later-derived catalytic function (Fig. 1). This scenario keeps information flow through evolution intact and does not require a substantial change of interfaces during evolution. For instance, the dsRNA template could be changed later in evolution to a dsDNA interface without conceptually affecting transcription of ssRNA. Also, mRNA could be added as the template for protein translation without needing a substantial change in the existing carriers of genetic information. In the same way, the replacement of dsRNA with dsDNA would leave existing interfaces (the generation of catalytic RNA and mRNA) intact. Thus, based on a simple engineering paradigm, it is proposed that dsRNA as the informational molecule was the first molecule to have evolved, and that catalytic ssRNA as the first ribozymes would be derived later in evolution.

## Testing the design-by-contract methodology: a mechanism for double-stranded origin of the genome

An engineering paradigm was used to predict the logical sequence of events that leading to the origin of the current genome where informational and catalytic properties are separated in two entities. This hypothesis can be tested by investigating whether the proposed functional steps can be translated into a mechanistic sequence of events. In this section, a gradual scenario for a double-stranded RNA origin of life is proposed that reflects the proposed functional scenario that was based on design-by-contract.

### From RNA oligomers to dsRNA

**Figure 2:**
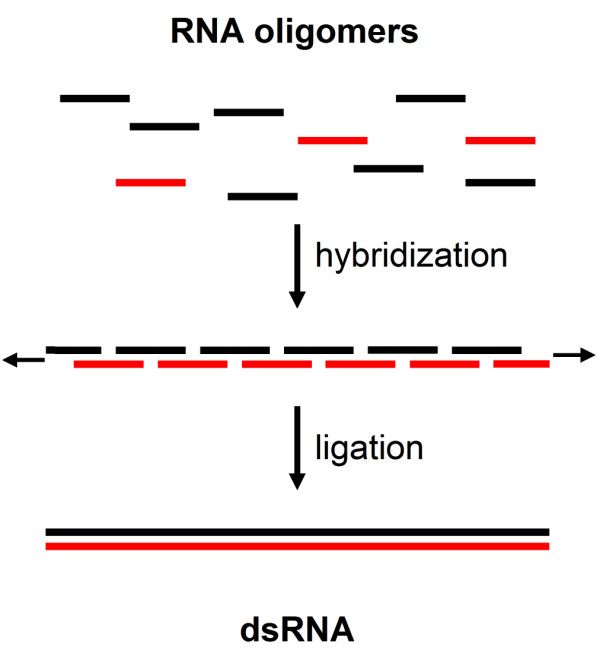
**Abiotic generation of dsRNA as a primitive information carrier**. Based on the availability of oligoribonucleotides, the first step in the generation of dsRNA is initiated when small oligonucleotides stick together to form an interrupted double-stranded chain of RNA. Ligation of the oligonucleotides by a slow abiotic process leads to a tight dsRNA chain.

The 'dsRNA first' hypothesis about the origin of life states that the first functional step was the appearance of dsRNA before the development of catalytic RNA and thus implies that dsRNA appeared abiotically. Assuming that short strands of RNA formed by template-directed abiotic ligation were available in a prebiotic world (for discussion see [16,17], dsRNA can be formed by the hybridization and ligation of complementary sequences of oligonucleotides (Fig. 2). In this process, oligonucleotides could initially built up an interrupted double-stranded chain of RNA, which was followed by a non-enzymatic ligation reaction to form an uninterrupted double-stranded piece of RNA. This mechanism is similar to the template-directed, non-enzymatic ligation and amplification of oligonucleotides [3,18-23] proposed to replicate an existing catalytic RNA molecule. Thus, the template-directed hybridization and extension of short oligonucleotides is a feasible approach for abiotic formation of longer strands of dsRNA.

### Replication by an abiotic chain reaction

**Figure 3:**
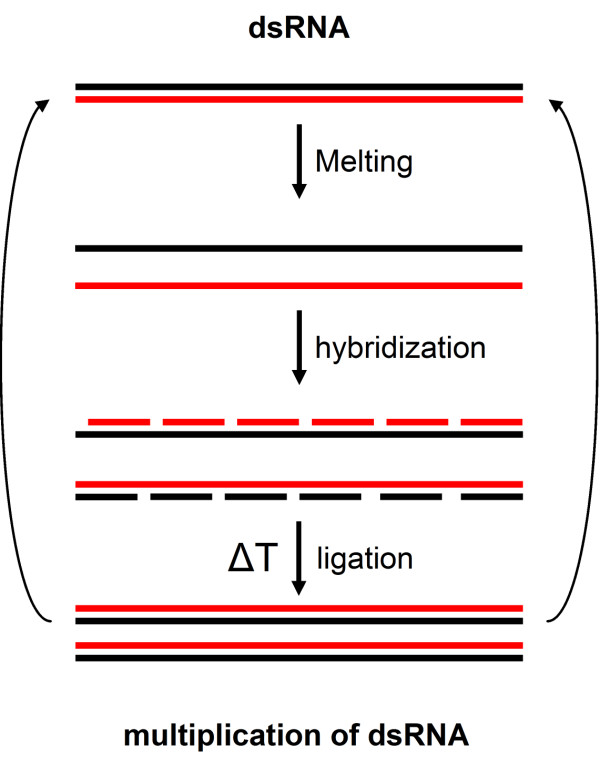
**Abiotical chain reaction to generate a pool of dsRNA**. Melting of dsRNA by an increase in temperature will results in two separate daughter strands. Upon lowering of the temperature, oligonucleotides can hybridize to the individual strands that are subsequently abiotically ligated to form a new chain of dsRNA. This dsRNA can then reenter the melting-polymerization chain, leading to an exponential increase or replication of the initial dsRNA strand. This process is similar to the polymerase chain reaction that amplifies dsDNA.

The crucial step in the origin of life is the formation of a replicating system that will allow genetic information to be transduced and amplified. The replication of dsRNA can be based on the intrinsic properties of complementary strands of ribonucleotides to form a stable double-stranded helix below the melting temperature of dsRNA and to separate into individual strands at temperatures above its melting temperature [24,25]. After temperature-induced strand separation, oligonucleotides can rehybridize to both individual strands upon lowering of the temperature to form new chains of interrupted dsRNA (Fig. 3). After non-enzymatic ligation of the interrupted strands (cf. Fig. 2), two new strands of dsRNA are formed. These newly formed dsRNA strands can then re-enter melting and rehybridization cycles, thereby amplifying the original strands of dsRNA. This process is similar to the polymerase chain reaction (PCR) used to amplify dsDNA, albeit with much slower polymerization time and could be driven by the diurnal cycle (see Discussion). The hybridization and extension of primer oligonucleotides to each other is frequently seen in PCR (e.g. [26-29] showing that thermal cycling in combination with ligation reaction can elongate as well as replicate oligonucleotides *in vitro*. Thus, using only non-catalytic short strands of ssRNA, dsRNA as an informational molecule can be formed and replicated abiotically.

### An early transcription mechanism

**Figure 4:**
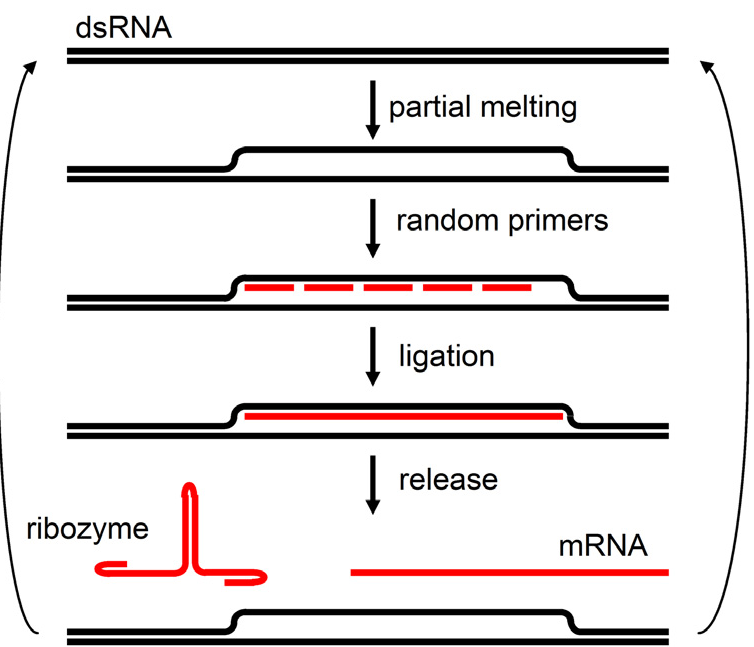
**Abiotic generation of the first catalytic RNA molecule**. The generation of ssRNA as a catalytic molecule can be accomplished by the partial melting of a double-stranded RNA helix, followed by the hybridization of RNA oligonucleotides to the resulting ssRNA strand upon lowering of the temperature. The hybridized oligonucleotides are then be annealed by a slow abiotic ligation process, similar to the one that is proposed in Figure 2 in the generation of dsRNA. The partial melting of the dsRNA, the rehybridization, and the release of the ssRNA can be accomplished by an oscillation in temperature.

The next step after the establishment of a pool of replicating dsRNA as a genetic information carrier is the subsequent generation of ssRNA that could function as a ribozyme (catalytic RNA; cf. Fig. 1). This generation of ssRNA from dsRNA can be accomplished by the partial melting of the double-stranded helix, followed by the hybridization of RNA oligonucleotides to the resulting partially separated strand (Fig. 4). The hybridized oligonucleotides can then be connected by a slow abiotic ligation process, basically similar to the one that is proposed in figures 2 and 3 in the generation and replication of dsRNA. This ssRNA can also be elongated in subsequent cycles since the partial sequence will selectively rehybridize with its anti-sense RNA, leading to a full size' transcript' in multiple ligation cycles. A subsequent release and folding of the hybridized RNA sequence from the template strand at higher temperatures would produce the first catalytic RNA (or later mRNA), a process that uses the same interface and is conceptually similar to enzyme-assisted transcription.

A change in temperature and/or salinity may cause a partial melting in regions of the dsRNA where the local melting temperature is lower due to the base composition. This partial melting or 'breathing' is a well-described phenomenon in dsDNA that occurs when thermal fluctuation opens up part of the dsDNA (passive opening) and allows components of the transcription machinery to bind the exposed single-stranded DNA [30,31]. Regions of RNA with specific nucleotide sequences (e.g. AU-rich) that have low local melting temperatures are more subject to partial melting and these specific regions could have represented early genes. Partial transcripts that rehybridize with partially melted RNA may also prevent the helix to close and thereby enhance their own transcription. Thus, the formation of an abiotic transcription bubble and subsequent abiotic transcription can be based on physico-chemical characteristics of dsRNA.

### From a dsRNA to a dsDNA world

**Figure 5:**
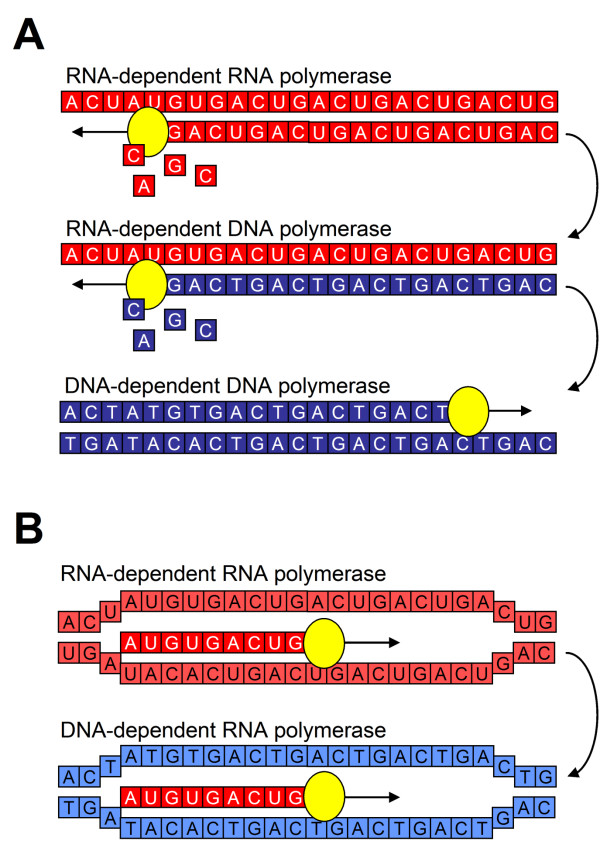
**Gradual transition from an RNA to a DNA world**. **A. **The transition from dsRNA to dsDNA can be accomplished by substituting RNA nucleotides for DNA nucleotides. This would require a switch in substrate-specificity from a RNA-dependent RNA polymerase to an RNA-dependent DNA polymerase, followed or accompanied by a mutation in the template specificity to a DNA-dependent DNA polymerase. **B. **The transcription of RNA from dsDNA is conceptually similar to transcription from dsRNA and this transition requires only a change in template recognition, i.e. from a RNA-dependent to a DNA-dependent RNA polymerase.

The advent of protein generation (translation) can speed up evolution by replacing existing abiotic replication and transcription processes by more efficient protein enzymes, for instance RNA polymerases. Based on the presence of existing dsRNA, in only a few gradual changes in an existing RdRp, the transition from a dsRNA to a dsDNA world can be made [4]. This transition is conceptually simple because it can be made by substituting the ribonucleotides building blocks of RNA with the deoxynucleotides of DNA (cf. Fig. 1). The insertion of deoxynucleotides by turning an existing RNA-dependent RNA polymerase (RdRp) into an RNA-dependent DNA polymerase RdDp) would effectively create a DNA-RNA hybrid, while the concomitant evolution to a DNA-dependent DNA polymerase (DdDp) would create dsDNA (Fig. 5A). The existing transcription process would only need a change in the template recognition site of an RdRp to a DdRp polymerase (Fig. 5B). Substrate specificity can be influenced by subtle modifications to a generic polymerase module [32] and most DNA polymerases are able to incorporate rNTPs as a substrate instead of dNTPs [33-36]. Also, template recognition of polymerases is not very specific and can be changed by minor mutations [37-40]. This would not have affected existing replication mechanism or involved the *de novo *development of a transcription machinery.

## Discussion

### Reverse engineering the origin of Life

The dsRNA first hypothesis proposes a sequence of functional events for the evolution of the genome that is derived from engineering principles that enforces functional continuity by keeping existing interfaces intact [14,15]. The dsRNA first hypothesis can be separated into distinct mechanistic and functional steps: a) the abiotic formation of dsRNA as a starting point for Life, b) the replication of this dsRNA in order to conserve and multiply the potential informational carrier, c) the formation of catalytic ssRNA from specific proto-gene regions of dsRNA, d) extension of functionality by protein translation based on a single-stranded RNA template (mRNA), and e) the transition from dsRNA to dsDNA. In this process, extra functionality is added in gradual steps while keeping existing functionality intact. The initial abiotic replication and transcription process can be exchanged for an enzymatic one, the evolution towards mRNA as a template for translation is only an addition to existing functions of ssRNA, while the transition from dsRNA to dsDNA does not require a basic change in functional interface. Thus, the dsRNA scenario maintains functional continuity in an evolving and expanding system, one of the fundamental requirements of evolution.

The engineering paradigm design-by-contract defined clear guidelines for evolution of developmental sequences that could be translated into a biochemical implementation. The presented scenario is also in line with other engineering considerations for a putative evolving molecular machine. The proposed mechanistic scenario provides a physical driving force for replication and transcription, the perpetual diurnal cycle, offering the possibility for cycles of extensions and mutations and a general trend towards an increase in complexity. Also, the scenario is self-contained because all processes later in evolution are derived from, and contained within the first (informational) dsRNA molecule. This makes evolution in its entirety independent, in contrast to genetic takeover scenario's. Thus, the dsRNA hypothesis provides an alternative theory for the origin-of-life that can be based on the predicted power of the framework and the resulting system characteristics, together with the gradual mechanistic scenario that maintains functional continuity.

### Microenvironments for early life

Although the dsRNA first hypothesis assumes the presence of short ribonucleotide strands or equivalents, the production of ribonucleotides still represents a challenge for prebiotic chemistry. A functional equivalent for RNA, for instance preRNA (see [41]), may substitute for the early abiotic processes that are suggested in this manuscript. The first formation of oligomers could have relied on a specific environment, for instance the surface of a catalytic mineral such as montmorillonite on which surface activated monomers could react to form short oligomers [42,43]. Some form of compartmentalization, for instance in liposomes or porous rock [44-46] may have provided the environment for early life when short strands of complementary oligonucleotides (see [17,42]) form in populations of abiotic microenvironments, for instance the pores of porous rock. Such abiotic microenvironments could later be functionally replaced by a cell membrane due to the evolution of lipid-generating enzymes [15]. This replacement of the abiotic environment provides more environmental independence while such a genome-driven membrane formation will also give the biological cell a self-contained character.

### Thermal cycling as the initial driving force for abiotic replication and transcription

The proposed melting and rehybridization cycles underlying the dsRNA-first hypothesis can be driven in a prebiotic environment by a diurnal cycle that amplifies and replicates populations of dsRNA. The perpetual night/day temperature cycles can in this view considered to be the driving force for the emergence of Life, creating an early potential for diversity and selection with each replication cycle. The thermal cycling PCR can in principle also occur in a steady temperature gradient where convection will cycle the DNA in and out of the higher temperature spot [47,48], widening the environments for an origin of life based on thermal cycling. Melting and hybridization steps are in principle also possible by tidal cycling [24] in combination with drying and increasing salt concentration, since increasing ionic strength also favors melting. The advent of biotic (enzymatic) replication and transcription process that replace the abiotic transcription processes would enable diurnal cycle independent evolution.

### Evolutionary drive towards formation of catalytic RNA

Similar to the tendency of ribonucleotides to hybridize and ligate to form dsRNA, a driving force towards ssRNA could cause the further transition to catalytic RNA in evolution. The abiotic transcription process would be specifically enhanced for proto-genes that generate a folded RNA. Since folded RNA's are less likely to rehybridize with its template in the abiotic transcription process, they avoid the product inhibition observed in other replicating systems [18]. Ribozymes and catalytic RNA are characterized by their folding into a three-dimensional structure like hairpin loops, and in combination with random mutation, a selection can take place for folded RNA with catalytic abilities. Selection of protocells in which ssRNA is generated that can specifically aid in the replication process may lead to selection for autocatalytic populations of dsRNA and may be considered to be proto-genomes. The emergence of ribozymes may also aid in the generation of genetic diversity by catalyzing splicing and ligation of RNA strands (cf. the role of introns; [14]).

### A design framework for evolution

Design-by-contract [49] forms a simple straightforward framework to design complex systems that need to be extensible and robust. Evolutionary steps can be modelled on this engineering paradigm by defining evolution as an expanding system of functionalities that are connected by well-defined and constant interfaces. The effect of designing interfaces across modules is a reduction of the interdependencies across modules or components and a reduction of the risk that changes within one module will create unanticipated changes in other modules. By identifying the functional modules and their conserved interfaces in evolution, it becomes possible to reconstruct the mechanistic scenarios underlying evolution. Here, an analysis of the interfaces for transcription and translation defined dsRNA as the logical first step towards an expanding genome. This approach led to a relative simple scenario for the origin of Life that makes a gradual scenario for genome evolution feasible. The same approach has recently also led to new scenario's for the origin of introns [14] and the origin of the eukaryotic cell [15] and shows the explanatory power of modelling evolution on design-by-contract and viewing Life as a self-evolving molecular machine.

## Competing interests

The author(s) declare that they have no competing interests.

## Reviewers' comments

### Reviewer's report 1

Anthony M. Poole, Stockholm University, Department of Molecular Biology & Functional Genomics, Stockholm, Sweden

This manuscript presents a highly speculative model for the origin of catalytic RNA via the prebiotic emergence of dsRNA, and includes a brief discussion of the RNA to DNA transition. I am not against speculation, but this area has been done to death, and in this particular case I do not feel the model sheds significant new light on the origin of life.

**Author's response: ***This article describes a predictive framework, in which evolutionary steps are derived from the developmental sequences. As such, the model is not speculative, but indicates a sequence of functional intermediates in he evolution of life and was tested by providing a mechanistic basis for the scenario. Moreover, the model also incorporates the principles of complex system design, such as robustness, flexibility and driving forces, and clearly exemplifies how the steps fit in a view of evolution as an expanding molecular machine. As far as I know, no predictive framework for early genome evolution exists and no gradual, functionally continuous, steps have been published leading to the current configuration of Life as we know it. I am sure that the engineering framework and the scenario itself will be interesting to many and hope that it will give a impetus to different origin-of-life research.*

I amended the article to stress the general setup of the article. I included the engineering methodology in the title and placed more emphasis on the functional continuity that is enforced by design-by-contract as a general framework for evolution. I also use more engineering terms related to complex system design including self-containedness and driving forces.

I will limit my criticism to three main points. Taking the latter stages first, the section concerning the RNA to DNA transition adds nothing new to discussions of the evolutionary origins of DNA. The author provides such a brief description of the RNA to DNA transition that it is not clear whether he favours the evolution of ribonucleotide reductases as a prerequisite to the origin of DNA. These enzymes perform the only known reaction leading to de novo deoxyribonucleotide synthesis (J Mol Evol 55:138 & 55:180). Furthermore, the growing consensus that the origin of DNA occurred very late in the evolution of cells (Biochimie 87:793; Mol Biol Evol 22:1444) is completely ignored. Explaining the origin of ribonucleotide reduction is non-trivial, as Forterre has recently argued (Biochimie 87:793). All the points regarding polymerase template and substrate specificity have been made in numerous other papers, and others have provided more in-depth discussion of the relevant experimental support for this. Entire papers have been devoted to specific parts of this complex transition; the current paper offers nothing new and is too patchy to be considered a concise summary of the existing body of work.

**Author's response: ***I show that the RNA to DNA transition is *functionally *simple, because for the system it would make no difference whether the mRNA is derived from a dsRNA or a dsDNA template (cf. double-stranded RNA viruses that are transcribed by DNA-dependent RNA polymerases. The biochemical transition is not trivial but this is a general question for evolutionary science and I refer to the literature mentioned. For my article, it is necessary that deoxyribonucleotide (and thus the evolution of ribonucleotide reductases) would be present as a substrate. The exact timing would be irrelevant and even a gradual or late implementation would be possible.*

As for the origin of polymerases, I do not know of any ribozyme counterpart or RNA constituent for any known polymerase, except for the RNA template of telomerase. Design-by-contract would predict that any RNA relics would be conserved in evolution in order to preserve existing interfaces, as is the case in transcription and translation. I therefore assume that RNA polymerase activity only evolved after protein translation, although RNA as a cofactor for abiotic ligation may be needed.

**Reviewer's response #2: **I think there is a risk that you may overstretch the predictive power you apportion to 'Design-by-contract'. In your paper, you say that there is no difficulty for dsRNA to be replaced by dsDNA, because this sort of change does not 'conceptually affect' any processes. We clearly see only partial preservation of RNA relics among modern cells (there are too few for us to see a complete RNA cell preserved within modern metabolism – see J Mol Evol 46:18), and some probable relics are not conserved in all three domains (making it harder to assign them to periods that date back to before the divergence of the three domains – see Curr Opin Genet Dev 9:672). More generally, replacements can occur without altering function, as exemplified by non-orthologous gene displacements (Trends Genet 12:334). These are important in the current case, because it would seem that this process must have happened in the origin of DNA genomes – perhaps more than once (Nucl. Acids Res. 27:3389; Mol. Biol. Evol. 22:1444). Given that interfaces remain intact in your scheme, but not necessarily the original functional units (i.e. DNA can replace RNA as genetic material), Design-by-contract cannot predict that anything as specific as RNA relics (i.e. a specific way of performing a function, tied to a specific type of chemistry) should be preserved, and therefore cannot be used to argue that ribozyme RNA polymerases never existed on account of them not currently existing. This point brings to mind Yarus' metaphorically charming 'Cheshire Cat' conjecture (FASEB J 7:31), and papers by Steitz which illustrate that a common two metal-ion mechanism for polymerization is at the basis of polymerization by unrelated polymerases, and is, chemically, within reach of catalytic RNA (e.g. Nature 391:231).

**Author's response #2: ***I agree that it is difficult to exclude the possibility that ribozyme RNA polymerases existed, and their functional replacement with protein counterparts would indeed be in agreement with design-by-contract. In developing the hypothesis, I did assume that the RNA relics we observe are caused by the difficulties in replacing them due the need for functional continuity, in line with design-by-contract. Therefore, I would have expected to see at least some representatives of ribozyme-containing RNA polymerases, as we see in transcription and translation. The lack of any RNA component for RNA polymerases has been one of the main drivers for developing the dsRNA hypothesis and represents one of the test cases for the use of design-by-contract.*

My next criticism is that the author ignores the considerable difficulties surrounding establishing plausible prebiotic syntheses for ribonucleotides. This amounts to what Joyce & Orgel have dubbed ' the molecular biologist's dream' – that these building blocks were readily synthesisable and available in sufficient amounts for the emergence of oligonucleotides (Joyce & Orgel Chapter 2 of The RNA world, 2nd edition. Gesteland, Cech & Atkins, eds. 1999 CSHL Press). The reasons why this is an unrealistic starting point for the origin of life have been expounded at great length, and, even considering current optimism surrounding possible prebiotic synthesis of ribose (e.g. Science 303:196), there is currently no reason to expect that abundant pools of nucleotides were available in prebiotic environments.

**Author's response: ***The scenario assumes the presence of the building blocks of life, but recognizes that this is not trivial. However, current prebiotic research is mainly focused on the abiotic formation of a single-stranded RNA molecule, and the present theory focuses on the generation of dsRNA as a start which may require completely different environments and reactions. For instance, hybridization of nucleotides and formation of strands of dsRNA may actually scavenge and protect single nucleotides. RNA cofactors and special microenvironments may facilitate abiotic ligation and provide specific hybridization conditions. In other words, I think that without a specific scenario to test, abiotic chemistry cannot a priori conclude that availability of oligonucleotides is an unrealistic starting point.*

We also seem to have a different definition of Life and its origin. My view on Life and its evolution is that of a self-contained system, forming basically a set of self-assembly instructions, and the origin of Life is about how this system formed. Anything that is not included in this system and part of the informational storage in the genome is in my view therefore a precondition for the origin of life, but it is not part of the system. Although the generation of nucleotides may not be simple, the essence of Life is our genome which is based on nucleotides and would therefore represent the starting point. If an abiotic generation of ribonucleotides is not possible and there are no functionally similar predecessors (e.g. preRNA), then an input from an external system seems the only other possibility. The dsRNA hypothesis for the origin of Life would not be different in such a case, and a discussion of such an environment beyond the scope of this article.

Finally, even if one elects to sidestep the 'prebiotic chemist's nightmare' and start at the 'molecular biologist's dream', for this kind of speculation to be useful, it needs to be backed up with experiments. The author should really specify some appropriate set of prebiotic conditions (temperature, salinity, oligonucleotide concentrations, etc.) and do experiments to show that, at minimum, dsRNA could be amplified under such conditions. Given that Dr. de Roos is arguing for a natural, non-enzymatic 'PCR-like' process driven by diurnal temperature changes, it would be straightforward to set up an experiment to examine whether such a system leads to ligation and subsequent amplification of dsRNAs from random RNA oligomers. That this system would lead to the emergence of catalytic RNA could likewise be experimentally tested. It is when one starts to entertain the experiments that one sees this model start to break down. For example, no explanation given as to why preformed ('prebiotic') RNAs would tend to prefer double-stranded intermolecular associations over intramolecular folding (given concentration of partners for dsRNA formation would presumably be low), nor why just catalytic ssRNAs would prefer folding into an active form rather than continuing to exist in the (according to the hypothesis presented) prevailing dsRNA state. Put simply, there is no reason why this should specifically hold for catalytic RNA as opposed to noncatalytic RNA – good folders are not necessarily good catalysts.

de Roos' model could be readily tested using existing experimental methods (a PCR machine for the thermal cycling required for non-enzymatic formation of dsRNA from oligonucleotides, and in vitro selection procedures to examine whether this process could lead to emergence of a simple catalytic RNA – here any catalytic RNA would suffice to convince me). This needs to be done before this idea can be taken seriously. My personal opinion is that if the author looks into the details of this more carefully, he will realise that the model as it stands is insufficiently detailed – and unrealistic.

**Author's response: ***I recognized and referred to the possibility for in vitro testing of this hypothesis, and mentioned that in the case of DNA, by-products of PCR could arise in the control situation when only primers are present. Also, the partial transcription processes could be tested in vitro. I do not however present this as a research article, but as a hypothesis based on theoretical considerations, and am hopeful that scientists that have access to the research tools may try these experiments.*

Folding of RNA is an important aspect and not trivial. I suggest that one of the driving forces towards catalytic RNA is folding since this will prevent rehybridization, but must be followed by natural selection for bioactive molecules. The sequence of the oligonucleotides determines folding, formation of dsRNA is dependent on temperature, salt concentration, sequence but short strand of ssRNA may also stabilize single-stranded regions. Therefore, I believe that there are enough possibilities for further research.

Finally, I recommend the interested reader to consider two papers by Paul & Joyce – the first reports a self-replicating ligase ribozyme, the second is a general review of this area (PNAS 99:12733 and Curr Opin Chem Biol 8:634). Paul & Joyce's experimental results do not solve the problems surrounding the origin of catalytic RNA, but they do provide an important piece of the puzzle, and illustrate how these problems are being productively addressed through experiment.

**Author's response: ***I generally read these articles with the following questions in mind. Is a framework applied that can be used to unravel the different steps in evolution? What mechanistic steps have been proposed to explain the origin of dsDNA as an information carrier and ssRNA as a catalytic molecule? Is there any physical evidence for the existence of a self-replicating replicase? How can functional continuity be guaranteed when moving from a single-stranded genome to a double-stranded genome? What are the driving forces for early evolution and how are the proposed different components reconciled in one genome? I believe that these questions should be addressed first because they are at the basis of an understanding of Life.*

I think the efforts in trying to make a self-replicating ligase ribozyme may be a dead-end for explaining the evolution of life. The theoretical approach used in my article not only identifies alternative solutions, it also argues against scenarios that violate the used engineering rules. The incorporation of a dual function in one molecule will not provide the necessary flexibility for the individual evolution of the different functionalities. It seems impossible to move from a single-stranded genome to a double-stranded while maintaining functional continuity. A reliance on external independent systems would not be in line with a required self-contained character of a complex system as evolution. Thus, before focusing on making a self-replicating replicase, we should be sure that it leads to an explanation of Life where these problems are sufficiently addressed to form a coherent mechanistic series of events.

### Reviewer's report 2

Eugene V. Koonin, National Center for Biotechnology Information, NIH, Bethesda, Maryland, United States

It seems to me that the central point of this proposal on the origin of first replicating systems is replication by thermal cycling (convectional PCR), possibly, with the involvement of ribozyme ligases, an activity that is, indeed, fairly easily selected in ribozymes. I think that this could have been made clearer in the subtract.

**Author's response: ***Thermal cycling was not supposed to be the central point of the article, but is used as an example how the predicted sequence of functional events can be implemented mechanistically and biochemically. The article predicts a sequence of events based on an engineering paradigm in which dsRNA as an informational carrier preceded catalytic ssRNA and served as a template for the generation of ssRNA. I have made this more clear in the article. See also comments to dr. Poole's review.*

Given the difficulties with the selection of highly processive ribozyme polymerases correctly pointed out by the author, this is an interesting idea. Incidentally, this possibility has been mentioned before, albeit in passing (Koonin, Martin, Trends Genet. 2005 Dec;21(12):647–54). This being said, the hypothesis is not developed into a coherent scenario for the origin of replicating ensembles of RNA or DNA molecules, with or without protein involvement. The difficulties with ribozyme polymerases are genuine, but it is unclear whether or not thermal cycling is capable of providing a viable alternative replication mechanisms and even less clear how the transition from this hypothetical primordial replication mechanism to a replication mechanism would occur. Further, I believe that the ease of the transition from dsRNA to dsDNA that the author finds so attractive is illusory given the major structural differences between dsRNA and dsDNA.

**Author's response: ***The main point of my article is the functional continuous scenario that is presented. As indicated in the article, the proposed transition from abiotic to biotic (enzymatic) replication is functionally similar, since the input and output are the same in both cases (dsRNA in, ssRNA out). I explained this in more detail in the comments to dr. Poole's review.*

The major structural differences between dsRNA and dsDNA do not prevent incorporation of ribonucleotides instead of deoxynucleotides by polymerases, hybrid strands to be able to form, or viral dsRNA to be transcribed by DNA-dependent RNA polymerases. Thus, it seems that the structural differences between dsRNA and dsDNA may have drastic effects on for instance stability, but preserve functional continuity. This is an important aspect in my theory: later modification of the informational carrier cannot affect existing functions.

Thus, although thermal cycling might have been one of the processes involved in the early evolution of life, I do not see any substantial explanatory power in the present hypothesis. The possibility of actual RNA replication by non-enzymatic thermal cycling is worth investigating experimentally, so, inasmuch as the present manuscript brings attention to such experiments, I find it useful. However, this is where it stops as there are no specific testable predictions here, and no coherent scenario for any of the stages in the early evolution of life.

**Author's response: ***As explained in the comments to dr. Poole's review, I use a predictive engineering framework that can be tested by investigating whether it is biochemically and biophysically feasible. I believe that the presented scenario is coherent in functional and in engineering terms. The mechanistic scenario can be tested in vitro as the figures give clear indications for real experiments, although I expect several modifications may be needed for the mechanistic scenario of the dsRNA-first scenario. As the article uses an engineering approach to model evolution, evolution could also be used as a test case to build an evolvable (software) system based on the same paradigms.*

### Reviewer's report 3

EugeneShakhnovich, Harvard University, Cambridge, MA, United States

This reviewer provided no comments for publication.
